# The Unitary Micro-Immunotherapy Medicine Interferon-γ (4 CH) Displays Similar Immunostimulatory and Immunomodulatory Effects than Those of Biologically Active Human Interferon-γ on Various Cell Types

**DOI:** 10.3390/ijms23042314

**Published:** 2022-02-19

**Authors:** Camille Jacques, Mathias Chatelais, Karim Fekir, Adrien Brulefert, Ilaria Floris

**Affiliations:** 1 Preclinical Research Department, Labo’Life France, 1 rue François Bruneau, 44000 Nantes, France; ilaria.floris@labolife.com; 2 ProfileHIT, 7 rue du Buisson, 44680 Sainte-Pazanne, France; mathias.chatelais@profile-hit.com (M.C.); karim.fekir@profile-hit.com (K.F.); 3 QIMA Life Sciences, 1 bis Rue des Plantes–CS 50011–86160, 44680 Gençay, France; adrien.brulefert@qima.com

**Keywords:** micro-immunotherapy, unitary medicine, interferon-γ, homeopathically prepared interferon-γ, centesimal Hahnemannian dilution, immunostimulant, immunomodulation, in vitro, immune-related markers, cytokines secretion

## Abstract

As a cytokine, gamma-interferon (IFN-γ) is considered a key player in the fine-tuned orchestration of immune responses. The extreme cellular sensitivity to cytokines is attested by the fact that very few of these bioactive molecules per cell are enough to trigger cellular functions. These findings can, at least partially, explain how/why homeopathically-prepared cytokines, and especially micro-immunotherapy (MI) medicines, are able to drive cellular responses. We focused our fundamental research on a unitary MI preparation of IFN-γ, specifically employed at 4 CH, manufactured and impregnated onto sucrose-lactose pillules as all other MI medicines. We assessed the IFN-γ concentration in the medium after dilution of the IFN-γ (4 CH)-bearing pillules and we evaluated in vitro drug responses in a wide range of immune cells, and in endothelial cells. Our results showed that IFN-γ (4 CH) stimulated the proliferation, the activation and the phagocytic capabilities of primary immune cells, as well as modulated their cytokine-secretion and immunity-related markers’ expression in a trend that is quite comparable with the well-recognized biological effects induced by IFN-γ. Altogether, these data provide novel and additional evidences on MI medicines, and specifically when active substances are prepared at 4 CH, thus suggesting the need for more investigations.

## 1. Introduction

The single member of the type II immune interferon family, gamma-interferon (IFN-γ), is a pleiotropic cytokine discovered in 1965, firstly identified as “macrophages-activating factor”, and today considered as a key immune mediator known to play an important role in innate and adaptative immune responses, including host antiviral/antibacterial defenses and antitumor activities. IFN-γ is indeed directly implicated in the regulation of a wide range of immune cells’ functions, as also in the control of their homeostasis and survival. For example, IFN-γ is an essential cytokine involved in macrophage activation and differentiation, in phagocytic cells’ activity, in T cell differentiation, activation and proliferation, in immune cell–cell communication, as well as in interactions between endothelial cells and immune cells [1]. Indeed, IFN-γ upregulates the gene expression of class I and II major histocompatibility complexes (MHC), which, in humans, are also called human leukocyte antigen (HLA) I and II [2], and it increases the expression of many other immune-related costimulatory molecules such as CD69 [3,4].

The immune cells that are recognized as the preponderant producers of this cytokine are natural killers (NK) and natural killer T (NKT) cells, as part of the innate immune response, and activated CD4 Th1 and CD8 cytotoxic T cells, as part of the adaptative/antigen-specific immune response [5]. The biologic effects of IFN-γ are mediated through its interaction with its dimeric receptor, namely IFN-γ receptor (IFNγR), comprising the IFNγR1 and IFNγR2 subunits [6], further ensuing the activation of the Janus kinase 1/2 (JAK1/2)-signal transducer and activator of transcription 1 (STAT1) signaling cascade [7]. On a molecular standpoint, the final steps of these pathways involved the translocation of the activated STAT1 homodimers to the nucleus and their binding to the γ-interferon-activated sites (GAS) family enhancers, resulting in the expression of the IFN-γ target genes. The most important effects of those IFN-γ’ activated genes are: (i) stimulation of NK cell and macrophages functions; (ii) increase of antigen-presenting molecules expression, including HLA molecules; (iii) activation of inducible nitric oxide synthase; (iv) promotion of immunoglobulins production from activated B cells; (v) induction of adhesion molecules expression required for leukocytes migration to the inflamed site [1,2,5,6,7].

Regarding its importance in both sides of the innate and the adaptative immune responses, the therapeutic potential of IFN-γ as an immunomodulatory drug has been evaluated in a wide variety of diseases, including viral and microbial infections of the respiratory tract such as pulmonary tuberculosis [8] and COVID-19 [9], but also in hepatitis, osteopetrosis as well as cancers [10]. Unglycosylated recombinant human-derived protein IFN-γ1b (Actimmune, InterMune) and replication-deficient adenovirus vectors expressing IFN-γ cDNA (TG-1041, TG-1042) are the two main forms of IFN-γ that have already been tested in clinical trials [11,12]. In spite of their therapeutic benefits for the patients, several side effects have been reported, including fever, headache, chills, asthenia, arthralgia, malaises, mild hematologic depression and transient hepatic enzyme abnormalities [13,14,15,16].

Interestingly, recent fundamental research on cytokine-mediated biological processes highlight the extreme cellular sensitivity to molecular stimuli and the fact that extracellular cytokine levels of only some femtomolar to picomolar were sufficient to properly trigger cellular responses. The case of the interleukin-6 (IL-6) is quite impressive, as a bioactivity study demonstrated that only four molecules of IL-6 per cell were significantly able to induce a cellular response, the latter even reaching its maximum with only eight molecules of IL-6 [17]. Considering these results and how one or very few molecules are able to activate a cell and/or an entire organism [18], the implications of those findings should be further considered in clinic and medicine, as they could bring all the therapeutic benefits of a molecule/component while minimizing its unwanted effects.

Regarding the implication of IFN-γ as a key mediator of the immune responses and its promising therapeutic effects, the inclusion of this agent into the medical arsenal at very low doses to induce effective responses while minimizing its side effects could be a great value for the patients. In this context, the use of homeopathically prepared cytokines deserves to be better considered and further studied, as it appears to have therapeutic potentials according to the currently available studies [19].

Micro-immunotherapy (MI) medicines are homeopathic medicinal products that include signaling molecules, such as cytokines, in their formulations [20,21,22]. All MI medicines have their composition expressed as centesimal Hahnemannian dilution (CH). The homeopathically prepared active substances are further impregnated onto sucrose-lactose pillules, also called globules, for an oromucosal administration. As an example of MI medicine that contains IFN-γ in its composition, the 2LEID^®^ can be cited, as it uses IFN-γ and TNF-α, in association with other immune regulators, to boost the immune system and different actors of the innate and adaptive responses. It has recently been shown that this medicine enhanced the proliferation and the activation of immune cells, as well as modulated the expression of several immune-related cell surface markers, thus acting as a promising immunostimulatory drug [23]. While IFN-γ is always combined with multiple signaling molecules in MI medicines, including, but not exclusively, the just-mentioned 2LEID^®^, we have investigated its biological effects as unitary medicine in several in vitro models.

In this fundamental research study, we are laying our investigations on the cellular immunomodulatory effects of one single homeopathically prepared cytokine at a time, human IFN-γ. We quantified the concentration of that cytokine, after dilution of the tested homeopathic product. While a quite high range of CH can be employed in MI medicines, or more generally in homeopathic products, our research focuses on the use of the unitary IFN-γ, at 4 CH. Discussion and investigation into the use of a specific amount of CH, and their specific effect, is outside the scope of this article. The research study aimed at providing novel preclinical evidence about the biological effects associated with its specific use, when prepared at 4 CH. In order to do so, several in vitro models of both primary and established cell lines were employed, such as primary immune cells isolated from healthy donors, including total peripheral blood mononuclear cells (PBMCs), granulocytes, CD14^+^-derived macrophages, or the human monocytic-derived THP-1 cell line, as well as non-immune cells, such as human endothelial cells.

## 2. Results

### 2.1. Few Picograms per mL of Human IFN-γ Are Contained in the Homeopathically Prepared IFN-γ (4 CH)-Impregnated Sucrose-Lactose Pillules

**Figure 1:**
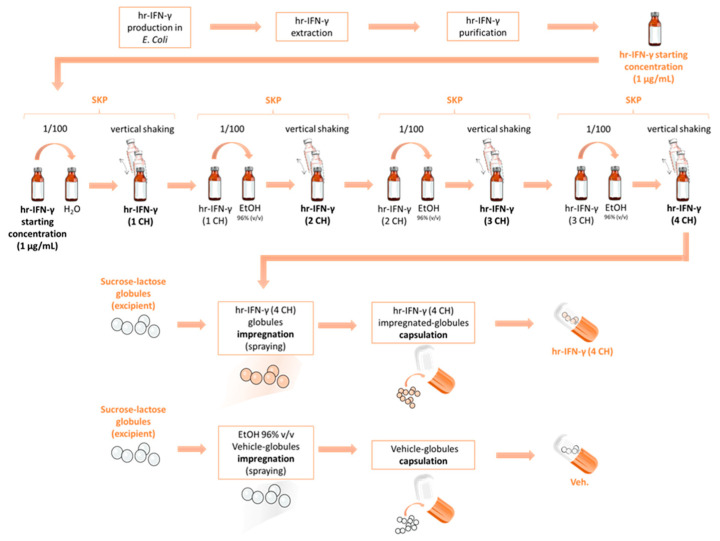
Representative scheme of the manufacturing process of the homeopathically prepared pillules of the vehicle-(Veh.) and the IFN-γ (4 CH) MI unitary. The Veh. pillules (or globules) are represented in white and the IFN-γ (4 CH)’s ones are represented in orange. The orange color used here is simply for the purpose of a better graphic understanding. The IFN-γ-impregnated pillules are white, so is the Veh., as the impregnation step does not change their appearance. hr: human recombinant; SKP: serial kinetic process.

The tested unitary MI medicine is a homeopathic product employing the human cytokine IFN-γ as active substance, with its composition expressed in CH, specifically at 4 CH. In order to better understand the specificities of the medicine, the manufacturing process (presented in Section 4) has been schematically represented in Figure 1. As illustrated, the initial starting concentration of the human IFN-γ (1 µg/mL) and the succession of the serial kinetic process (SKP)-steps leads to the final 4 CH dose further employed.

**Figure 2:**
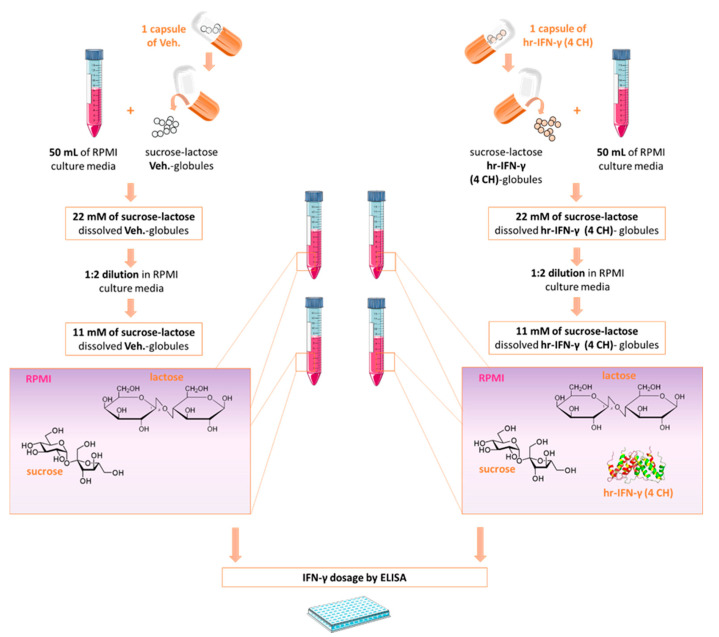
Few picograms per mL of human IFN-γ are contained in IFN-γ (4 CH)-impregnated sucrose-lactose pillules. Schematic representation of the globules’ dilution process within the culture media and the experimental protocol. IFN-(4 CH)-pillules and Veh.-pillules were diluted in RPMI medium to reach the sucrose-lactose concentrations of 11 mM and 22 mM. The concentration of human IFN-γ was assessed by ELISA either right after the dilution (fresh dilution). hr: human recombinant. Modified from https://commons.wikimedia.org/wiki/File:Interferon_Gamma.png, accessed on the 25 January 2022.

With the aim of investigating and quantifying the presence of human IFN-γ, the two types of pillules, the Veh.- and the IFN-γ (4 CH)-impregnated sucrose-lactose pillules, were freshly diluted within the culture media, to reach the sucrose-lactose concentrations of 11 mM and 22 mM (Figure 2). An ELISA kit having a detection limit of 1.04 pg/mL for human IFN-γ was chosen to investigate the presence of the active in the medium, after dilution of the IFN-γ (4 CH) pillules.

As expected, undetectable levels (<1.04 pg/mL) of human IFN-γ were found in the Veh. control-diluted containing medium, at both concentrations of the tested pillules. The results describing the measured levels of human IFN-γ for the tested unitary MI medicine are summarized in Table 1. The mean concentrations of IFN-γ, the standard deviations (SD), the medians and the maximal (Max) values (all expressed in pg/mL), obtained for each of the two tested concentrations, have been reported in Table 1. Thus, the sucrose-lactose pillules contained in one capsule of IFN-γ (4 CH), when diluted in 50 mL of RPMI medium, contain about 2 pg/mL of human IFN-γ.

This experiment has been performed once, so no statistical inference has been done. Even if those results should still be confirmed, they provide, for the first time, information about the presence of an active substance within the pillules used in MI, and allow us to establish a correlation between the amount of IFN-γ in pg/mL and its dosage in CH, after having been homeopathically prepared through the SKP.

### 2.2. In Vitro, IFN-γ (4 CH) Increases the Proliferation and the Activation of Granulocytes and It Enhances the Expression of HLA-DR in Monocytes/Macrophages

**Figure 3:**
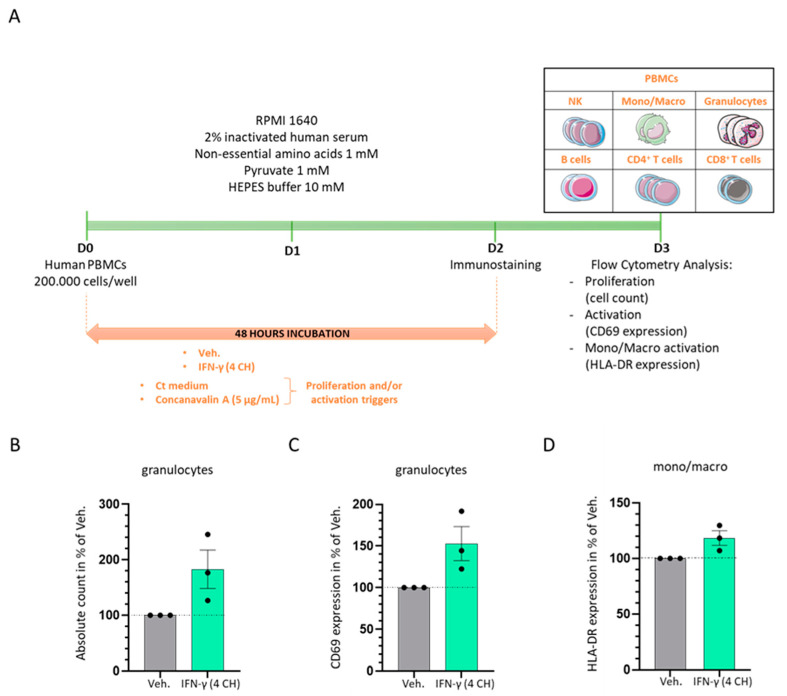
IFN-γ (4 CH) increases the proliferation and the activation of granulocytes and the expression of HLA-DR of monocytes/macrophages in vitro. (**A**) Representative scheme of the experimental protocol. Human PBMCs from three healthy donors were cultured during 48 h in classical culture conditions in presence of IFN-γ (4 CH)/Veh. The cells were immune-stained at D2 and analyzed by flow cytometry at D3. The total number of cells, the total cell count within each subpopulation (NK cells, monocytes/macrophages, granulocytes, B cells, T cells, CD4^+^ T cells, CD8^+^ T cells) and their activation status were assessed. Each cell subpopulation was discriminated according to the expression of the markers referred in the Material and Methods section. The expression of the CD69 marker was evaluated in each subpopulation as the activation marker of reference. The expression of HLA-DR was assessed within the monocytes/macrophages subpopulation as a supplementary activation marker. (**B**) The total number of granulocytes was evaluated. Grey-shaded histograms represent the vehicle (Veh.) and green-shaded histograms represent IFN-γ (4 CH) treatment. Each histogram represents the mean ± SEM of the cell count obtained for each individual donor as a percentage of the IFN-γ (4 CH)-treated-condition count normalized to the Veh-condition. Each individual point (black dot) represents the average of a triplicate per donor. (**C**) IFN-γ (4 CH) induces the expression of CD69 in granulocytes. Each histogram represents the mean ± SEM of the CD69 expression obtained for each individual donor as a percentage of the IFN-γ (4 CH)-treated-condition CD69 expression normalized to the Veh-condition. Each individual point (black dot) represents the average of a triplicate per donor. (**D**) IFN-γ (4 CH) induces the expression of HLA-DR in monocytes/macrophages (mono/macro) cells. Each histogram represents the mean ± SEM of the HLA-DR expression obtained for each individual donor as a percentage of the IFN-γ (4 CH)-treated-condition HLA-DR expression normalized to the Veh-condition. Each individual point (black dot) represents the average of a triplicate per donor.

The in vitro experiment was planned to address the capabilities of IFN-γ (4 CH) to stimulate the proliferation and the activation of human PBMCs in a “naïve” basal state. As illustrated in Figure 3A, human PBMCs freshly isolated from three healthy donors were cultivated in presence of IFN-γ (4 CH) or Veh. during 48 h in classical culture conditions. Total cells and the different immune cells’ subpopulations depicted in the representation in Figure 3A (NK cells, monocytes/macrophages, granulocytes, B lymphocytes (B cells), T lymphocytes (T cells), CD4^+^ T cells and CD8^+^ T cells) were counted by flow cytometry and their activation status was monitored through their CD69 expression levels. The results presented in Figure 3B,C show that IFN-γ (4 CH) was able to induce and almost double the proliferation of granulocytes and also remarkably increase their activation. Data of the cell counts and the CD69 expression obtained for each individual donor are presented in Supplementary Figure S1A,B. The effect on the other cell populations was smaller; we observed an increase of the proliferative capacities in IFN-γ (4 CH)-treated NK cells, T cells, CD4^+^ T cells and CD8^+^ T cells (of about 10% more than the vehicle-treated cells) (data not shown), while no effect was found on their CD69 expression levels. We also wanted to assess the effect of IFN-γ (4 CH) on the expression of HLA-DR in the monocytes/macrophages’ population, in those basal conditions. As represented in Figure 3D, HLA-DR expression was increased in about 20% in IFN-γ (4 CH)-treated cells compared with the vehicle-treated ones. Data of the HLA-DR expression obtained for each individual donor are presented in Supplementary Figure S1C. As the experiment has only been performed once, the data presented here were not subjected to inferential statistics. Notwithstanding, it can be concluded that IFN-γ (4 CH) may have proliferative and activation enhancing capabilities towards the cells of the innate side of the immunity, such as granulocytes and macrophages.

### 2.3. IFN-γ (4 CH) Stimulates Granulocytes Phagocytosis Capabilities In Vitro

**Figure 4:**
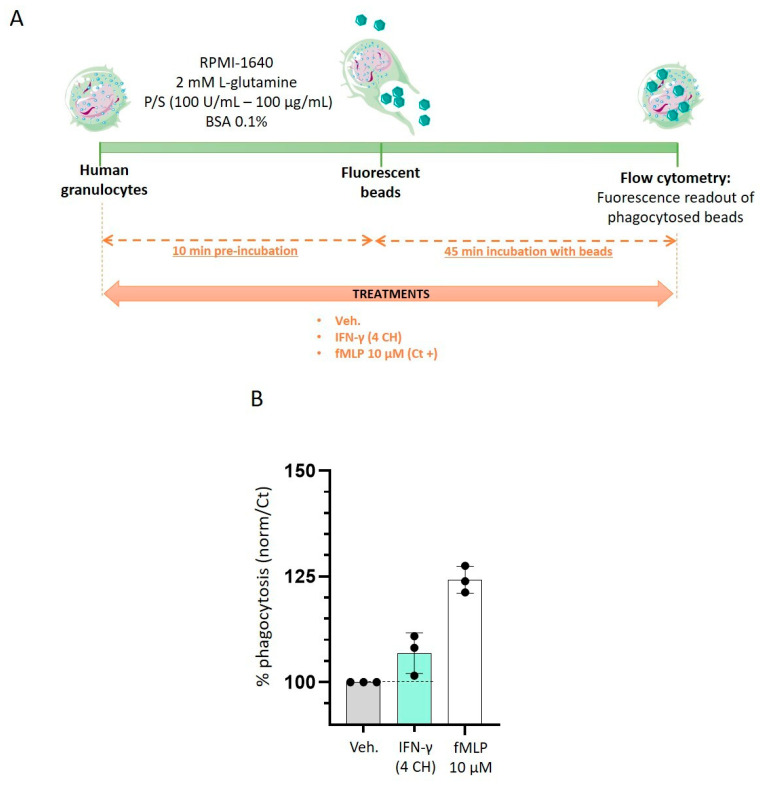
IFN-γ (4 CH) stimulates the phagocytosis capabilities of granulocytes. (**A**) Schematic representation of the experimental protocol. Human granulocytes were preincubated during 10 min with IFN-γ (4 CH), or vehicle (Veh.), or 10 µM N-formyl-methionyl-leucyl-phenylalanine (fMLP) as a positive phagocytosis inducer. Fluorescent beads (represented in green) were then added to the culture medium for an additional 45 min. The cells were then rinsed and the fluorescence was assessed by flow cytometry. P/S: penicillin/streptomycin; BSA: bovine serum albumin. (**B**) IFN-γ (4 CH) stimulates the phagocytosis’ capabilities. Each condition was performed in triplicate and each histogram represents the mean ± SD of the fluorescence as a percentage of the vehicle-treated conditions, set at 100%. The black dot line was drawn to better visualize in the graph the effect of MIM compared to its Veh. control.

Having observed the enhanced granulocytes’ proliferation and activation in IFN-γ (4 CH)-treated cells, we wanted to assess the effects of this factor on their phagocytosis capabilities. As depicted in Figure 4A, human granulocytes were thus preincubated during 10 min with either Veh. or IFN-γ (4 CH). After the preincubation time, fluorescent beads were added to the medium and the cells were incubated for another 45 min. The phagocytosis was assessed by flow cytometry, and the normalized results were expressed as a percentage of the vehicle-treated cells, set at 100% (Figure 4B). In parallel, in order to include a positive control in the experiment, the cells have also been treated with 10 µM of N-formyl methionyl-leucyl-phenylalanine (fMLP), regarding the phagocytosis-inducing capabilities of this compound [24]. Our data show that IFN-γ (4 CH) increased the phagocytosis by about 10% compared with the Veh. control group (Figure 4B). While these results should be substantiated by performing this experiment again, they are still encouraging, as they highlight the effect of IFN-γ (4 CH) in stimulating the granulocytes’ function, reinforcing their phagocytic capacities.

### 2.4. IFN-γ (4 CH) Increases the TNF-α Secretion in CD14^+^-Derived Macrophages and THP-1 Cells, and It Modulates the Cytokines’ Secretion Profile in CD14^+^-Derived Macrophages

**Figure 5:**
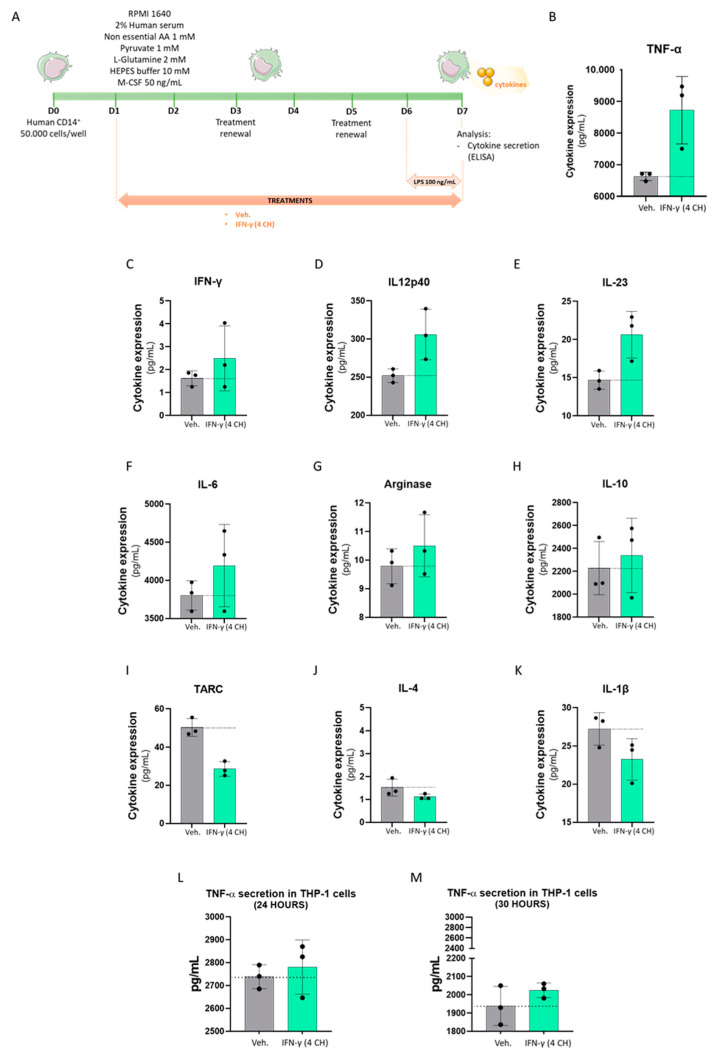
IFN-γ (4 CH) upregulates the secretion of TNF-α in LPS-inflamed CD14^+^-derived macrophages and in THP-1 cells and it modulates the cytokines’ secretion profile in CD14^+^-derived macrophages. (**A**) Representative scheme of the experimental protocol. CD14^+^ cells were obtained from PBMCs isolated from one healthy donor and cultivated during 6 days in complete medium supplemented with M-CSF 50 ng/mL (M0) and in the presence of IFN-γ (4 CH) or vehicle (Veh.) Additional 24 h of LPS treatment (100 ng/mL) were applied as an inducer of an inflammatory status during the last day of the IFN-γ (4 CH)/Veh. treatment. AA: amino acids. Cytokine secretion was evaluated by ELISA and the cell surface markers’ expression was assessed by flow cytometry. Effect of IFN-γ (4 CH) on the secretion of the cytokines: TNF-α, IFN-γ, IL12p40, IL-23, IL-6, arginase, IL-10, TARC, IL-4, and IL-1β (**B**–**K**). THP-1 cells, obtained from ATCC (Manassas VA, USA), were cultured in RPMI 1440 supplemented with 10% fetal calf serum (FCS) and antibiotics. For the experiment, THP-1 cells were pre-treated with IFN-γ (4 CH) or Veh. for 30 min, and stimulated with LPS (1 μg/mL) for 24 and 30 h (**L**–**M**). The supernatants were collected and stored at −80 °C until use. Then, TNF-α was measured by ELISA. The two graphs represent the secreted levels in pg/mL. The mean ± SD are represented. In all the presented graphs, black dot lines were drawn to better visualize the effect of MIM compared to its Veh. control.

Given the fact that IFN-γ is a well-known inducer of macrophage-polarization towards a M1 profile [25], we then wanted to assess if IFN-γ (4 CH) could modulate the cytokine secretion profile and the expression of macrophages’ cell surface markers in a manner which could reflect their specific polarization profile, functionally induced by IFN-γ at higher doses. Freshly isolated human CD14^+^ cells were thus cultivated in basal M0 conditions (complete medium + M-CSF 50 ng/mL), in the presence of either the Veh. or IFN-γ (4 CH) for 6 days (Figure 5A). In order to reveal the cytokine secretion, a strong pro-inflammatory signal was applied to the cells, in the form of lipopolysaccharide (LPS) treatment (100 ng/mL). This stimulation was implemented during the last 24 h of the Veh./IFN-γ (4 CH) treatment (Figure 5A), prior to the ELISA assay. Cell viability was controlled before further analysis and IFN-γ (4 CH) did not present any toxicity on the CD14^+^-derived macrophages (data not shown). The secretion of tumor necrosis factor-α (TNF-α), IFN-γ, interleukin-12 subunit 40 (IL12p40), interleukin-23 (IL-23), interleukin-6 (IL-6), arginase, interleukin-10 (IL-10), thymus- and activation-regulated chemokine (TARC), interleukin-4 (IL-4) and interleukin-1β (IL-1β) was assessed, and the results are presented in Figure 5B–K. Even if the statistical significance of these results has not been tested, the secretion of TNF-α was importantly upregulated by IFN-γ (4 CH) treatment (of about 2000 pg/mL, corresponding to a 30% induction compared to the Veh.) (Figure 5B). As Figure 5C shows, the levels of IFN-γ are just slightly higher (about 1 pg/mL) in IFN-γ (4 CH)-treated cells compared to Veh.-treated cells. Considering the results presented in Table 1, more specifically those concerning the pillules concentration of 11 mM, it can be deduced that the increase of IFN-γ observed in IFN-γ (4 CH)-treated cells is probably due to the presence of the active substance in the medium, instead of an effect induced by the treatment itself, on cytokine secretion. Then, the secreted levels of IL12p40, IL-23, IL-6, arginase and IL-10 were slightly upregulated by IFN-γ (4 CH) (Figure 5D–H), while the one of TARC, IL-4 and IL-1b were slightly downregulated (Figure 5I–K), compared with the vehicle-treated cells.

Human monocytic THP-1 cells, extensively used for studying the immune response capacity of monocytes and monocyte-derived macrophages, have the ability to produce and secrete cytokines in response to LPS (1 μg/mL) [26], similarly to primary PBMC-derived macrophages [27]. For this reason, in order to confirm the findings and include one more in vitro model, THP-1 cells were cultivated with IFN-γ (4 CH) or Veh. in combination with LPS for 24 and 30 h. Then, the secretion of the pro-inflammatory cytokines TNF-α and IFN-γ has been investigated. As expected, LPS increased remarkably the levels of secreted TNF-α after 24 and 30 h, while IFN-γ was not detected by the ELISA. Figure 5L,M shows the results of the tested IFN-γ (4 CH) and its control (Veh.) It can be observed that, after both 24 and 30 h of treatment, IFN-γ (4 CH) slightly increased the secreted levels of TNF-α corresponding to about 40 pg/mL after 24 h (Figure 5L) and to about 85 pg/mL after 30 h (Figure 5M). Although the magnitude of effects is quite lower compared to the one reported in CD14^+^ cells for the same cytokine, on the whole, the results are consistent. Once again, these experiments have only been performed once and inferential statistics were not performed. However, these results tend to demonstrate that IFN-γ (4 CH) could have a stimulatory effect on the secretion of a panel of pro-inflammatory cytokines, and especially TNF-α, in both human monocytic cell line and human primary monocytic-derived macrophages, under LPS exposure.

### 2.5. IFN-γ (4 CH) Modulates the Expression of Membrane Markers in an In Vitro Model of CD14^+^-Derived Macrophages

**Figure 6:**
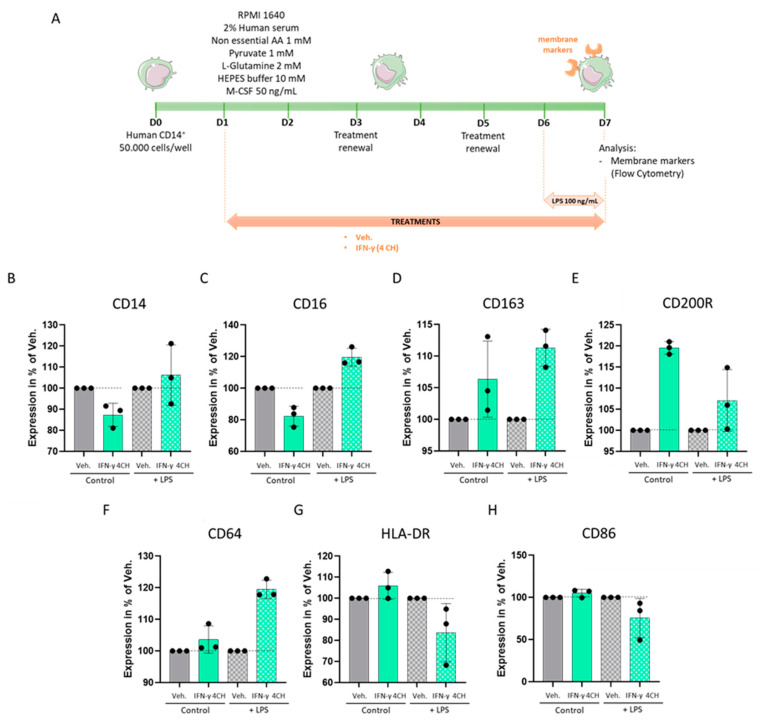
IFN-γ (4 CH) modulates the expression of membrane markers in an in vitro model of CD14^+^-derived macrophages. (**A**) Representative scheme of the experimental protocol. CD14^+^ cells were obtained from PBMCs isolated from one healthy donor and cultivated during 6 days in complete medium supplemented with M-CSF 50 ng/mL (M0) and in the presence of IFN-γ (4 CH) or vehicle (Veh.) An additional 24 h of LPS treatment (100 ng/mL) were applied as an inducer of an inflammatory status during the last day of the IFN-γ (4 CH)/Veh. treatment. Cell surface markers’ expression was assessed by flow cytometry. AA: amino acids. Effect of IFN-γ (4 CH) on the expression of the cell surface markers: CD14, CD16, CD163, CD200R, CD63, HLA-DR, and CD86 (**B**–**H**), in presence (+LPS) or not (Control) of LPS. The mean ± SD are represented. Plain-colored histograms represent the control conditions (w/o LPS), whereas squared-patterned histograms delineate the LPS conditions. In all the presented graphs, black dot lines were drawn to better visualize the effect of MIM compared to its Veh. control.

As IFN-γ (4 CH) seemed to enhance the secreted levels of pro-inflammatory cytokines in CD14^+^-derived macrophages; we then wanted to evaluate if it could also modulate the expression of immune-related membrane markers. The expression levels of seven macrophages’ surface markers (CD14, CD16, CD163, CD200R, CD64, HLA-DR and CD86) were thus assessed by flow cytometry. In order to appraise if the LPS stimulation was used to induce the cytokine secretion in the previous results’ section also had an impact on the cell surface markers, the experiment was performed with (w) and without (w/o) LPS-priming (scheme of the experimental protocol in Figure 6A), and the results are presented Figure 6B–H. Several variations of expression patterns seemed to emerge depending on the marker and the presence/absence of LPS, characterized by either (i) expression’s downregulations w/o LPS-priming/expression’s upregulations w LPS-priming; (ii) similar expression’s upregulations in both conditions (w and w/o LPS); or (iii) expression’s upregulations w/o LPS/expression’s downregulations w LPS-priming. For example, w/o LPS-priming, IFN-γ (4 CH) tends to slightly decrease both CD16 and CD14 expression after 6 days of treatment, compared with the Veh. alone, whereas an increase in the expression of these two markers was observed in the case of the IFN-γ (4 CH)-LPS-primed cells (pattern i, in Figure 5C and Figure 6B). The expression levels of CD163, CD200R, and CD64 were all three upregulated by IFN-γ (4 CH) compared with the Veh. in both conditions (pattern ii, in Figure 6D–F). Finally, w/o LPS-priming, IFN-γ (4 CH) induced a slight tendency towards an increase in the expression of HLA-DR and CD86 compared to the Veh., whereas it downregulated their expression in an LPS-treated context (pattern iii, in Figure 6G,H). All these results, while derived from experiments performed once, provide first-time information about the effects of a low dose IFN-γ (4 CH) on the immune-related cell membrane markers of CD14^+^-derived macrophages, with or without LPS, the latter context being transposable to bacterial-infection conditions in humans.

### 2.6. IFN-γ (4 CH) Enhances the Activation of CD3-Pre-Primed Immune Cells In Vitro

**Figure 7:**
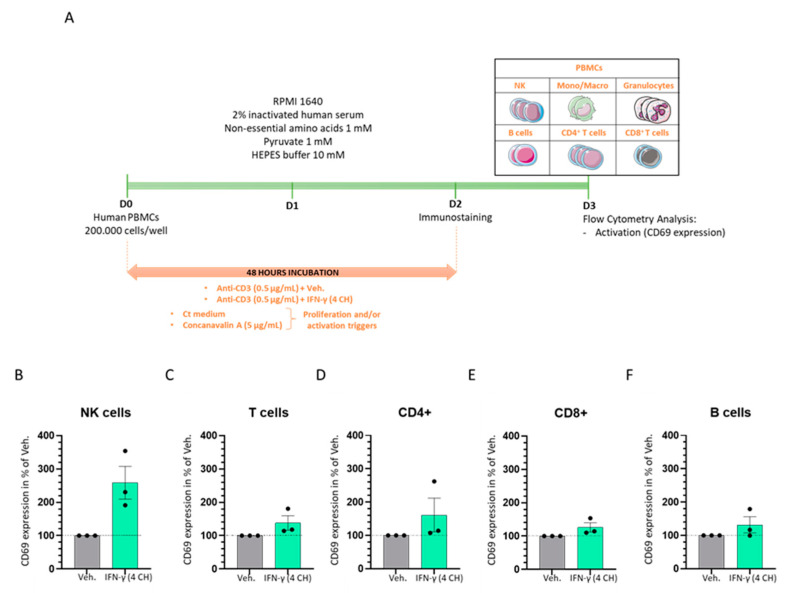
IFN-γ (4 CH) enhances the activation of CD3-pre-primed immune cells in vitro. (**A**) Representative scheme of the experimental protocol. Human PBMCs from three healthy donors were cultivated during 48 h in classical culture conditions in presence of anti-CD3 (0.5 µg/mL) plus IFN-γ (4 CH)/Veh. The cells were immuno-stained at D2 and their activation status, characterized by the CD69 expression, was analyzed by flow cytometry at D3. Each cell subpopulation (NK cells, monocytes/macrophages, granulocytes, B cells, T cells, [CD4^+^ and CD8^+^]) was discriminated according to the expression of the markers referred in the Material and Methods section. The CD69 expression was assessed within the different subpopulations: NK cells, T cells, CD4^+^ T cells, CD8^+^ T cells and B cells (**B**–**F**). Each histogram represents the mean ± SEM of the CD69 expression obtained for each individual donor as a percentage of the IFN-γ (4 CH)-treated-condition CD69 expression normalized to the Veh-condition. Each individual point (black dot) represents the average of a triplicate per donor. In all the presented graphs, black dot lines were drawn to better visualize the effect of MIM compared to its Veh. control.

In order to assess whether IFN-γ (4 CH) could differentially modulate towards an increase or a decrease, the activation status of CD3 pre-primed cells, PBMCs from three healthy donors were incubated during 48 h in presence of anti-CD3 antibody, plus either the Veh. or IFN-γ (4 CH) (Figure 7A). The cells were then immuno-stained and the NK cells, granulocytes, T cell (CD4^+^ and CD8^+^) and B cell subpopulations were discriminated thanks to the flow cytometry technique, based on the expression of the markers’ panel described in the Material and Methods section. As previously described for the PBMCs cultured under basal naïve standard conditions, the CD69 marker allowed us to evaluate the activation level of each subpopulation in response to IFN-γ (4 CH). The overall results, presented in Figure 7, highlighted that all the immune cells subpopulations responded to such treatment with an increase of their activation status, compared with the Veh. control. In particular, NK cells displayed the highest effect in terms of magnitude, with an increase in the CD69 expression of about 150% (Figure 7B). Then, according to the order of magnitude of the effect compared to the vehicle-treated cells, CD4^+^ T cells showed an increase of about 60% (Figure 7C); the total T cells, of about 40% (Figure 7D); the CD8^+^ T cells and the B cells, of about 30% (Figure 7E,F) of their respective CD69 expression. Data of the CD69 expression in each of the above-mentioned subpopulations obtained for each individual donor are presented in Supplementary Figure S2. As it is legitimately established that a wide variability of results exists within cellular models of primary cells isolated from patients, we are well aware that those results should be confirmed with more donors. However, the trends delineated here seem to support the role of IFN-γ (4 CH) as a proper inducer of the PBMC subpopulation activation in a CD3-primed environment. They also presume the role of this factor as an additional co-stimulatory signal, even at the low dose of 4 CH, as used and manufactured in the context of MI.

### 2.7. Commercially Available Human Recombinant IFN-γ and Homeopathically Prepared IFN-γ (4 CH) (Each Compared to Their Own Controls) Have Both DownRegulated the Expression of the Four Tested Endothelial Cell Surface Markers In Vitro

**Figure 8:**
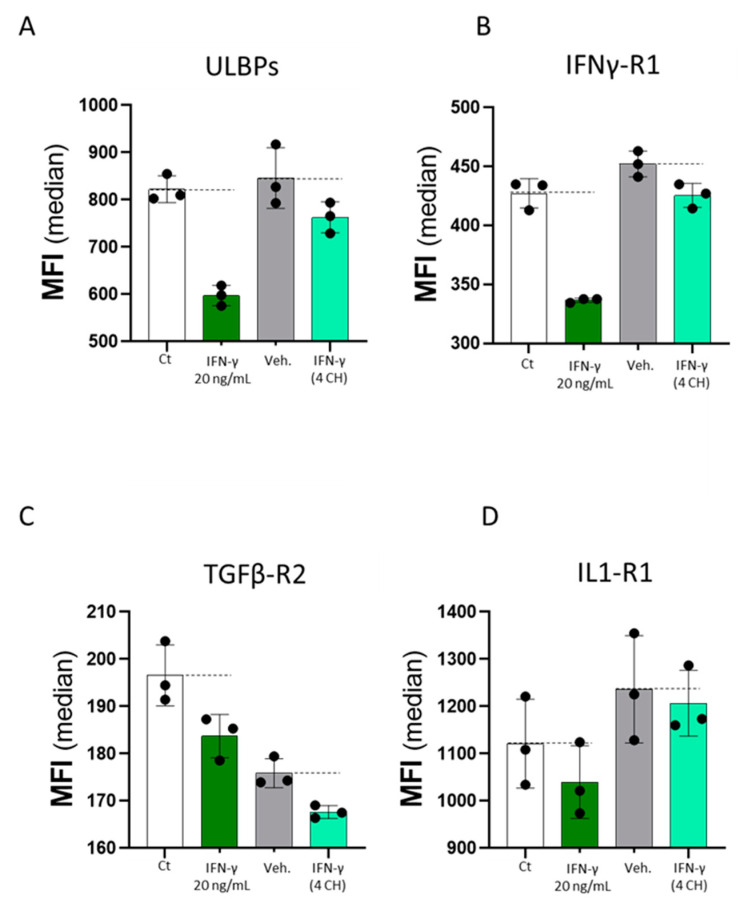
Commercially available human recombinant IFN-γ (20 ng/mL) and homeopathically prepared IFN-γ (4 CH), each compared to their own controls, have both decreased the expression of the four tested immunity related endothelial cell surfaces. HUVECs were incubated during 48 h in the presence of IFN-γ (4 CH) or the vehicle (Veh.) The only medium was run as a negative control, while IFN-γ at 20 ng/mL was used as a positive control of the expected biological effects of IFN-γ. IFN-γ (20 ng/mL), as well as IFN-γ (4 CH), both decrease the expression of the four tested endothelial cell surface markers: ULBPs, IFNγ-R1, TGFβ-R2, and IL1-R1 (**A**–**D**). The results represent the mean ± SD of one technical triplicate for each condition. In order to well-read and interpret the graphs, we drawn two dotted lines per graph at the mean levels of either the medium control (Ct) or the Veh. control to observe and estimate the magnitude of effect induced by IFN-γ (20 ng/mL) or by IFN-γ (4 CH), respectively. In all the presented graphs, black dot lines were drawn to better visualize the effect of MIM compared to its Veh. control.

Finally, as IFN-γ (4 CH) seemed to display direct effects on the expression of immune cells’ activation-related surface markers, we were wondering if these features could also be extended to endothelial cells, as those cells are right at the interface between the circulation and the local immune reaction within the organism. In order to investigate the biological effects of IFN-γ on these cells, commercially available recombinant human IFN-γ used at 20 ng/mL, or homeopathically prepared IFN-γ (4 CH), under the form of the unitary MI medicine, were employed to treat primary human umbilical vein endothelial cells (HUVECs). The cells were thus cultivated with the two tested items for 48 h, and additional controls, made of cells cultivated with either the culture media only, or the vehicle alone (Veh.) were used as respective controls of the two tested IFN-γ versions. In an attempt to compare if both forms of IFN-γ can similarly or differentially modulate the expression of immune-related cell membrane markers, the expression of four cell surface markers was evaluated by flow cytometry: UL16 binding proteins (ULBPs), interferon-γ receptor 1 (IFNγ-R1), transforming growth factor-β receptor 2 (TGFβ-R2) and interleukin-1 receptor 1 (IL1-R1). IFN-γ (20 ng/mL) induced the repression of the four surface markers (IFNγ-R1, ULBPs, IL1-R1, TGFβ-R2) respectively in about 25%, 25%, 10%, and 5% compared to the untreated control (Ct). Similarly, as shown in Figure 8, IFN-γ (4 CH) was able to reduce the expression of the four analyzed markers compared to its Veh. control, too. To evaluate the magnitude of the effect, the expression levels observed in IFN-γ (4 CH)-treated HUVECs were compared with the proper experimental control, Veh.-treated HUVECs and the effect, expressed in % vs. Veh., was of about 10%, 6%, 5% and 2%, respectively for ULBPs (Figure 8A), IFNγ-R1 (Figure 8B), TGFβ-R2 (Figure 8C) and IL1-R1 (Figure 8D). No statistical inference was performed on this body of data, as the previous experiment has only been completed once. However, as a whole, these results may highlight similarities in the IFN-γ pattern of effects regarding its endothelial cells’ markers expression modulative capabilities, when the cells are treated with either its commercially available human recombinant version, or its homeopathically prepared form, at 4 CH, independent of any other stimulus.

## 3. Discussion

We demonstrated here that the unitary MI 4 CH-homeopathically prepared human IFN-γ (see the description of its preparation in Section 4 and in the illustration in Figure 1) contains few pg/mL of active substance, at the two tested sucrose-lactose pillules concentrations (11 and 22 mM, corresponding to the content of one capsule diluted in 100 mL and 50 mL of medium, respectively) (see Table 1). It is therefore interesting to mention that basal circulating levels of IFN-γ have been reported to sit at a median of 5 pg/mL in healthy children. These data suggest that the observed IFN-γ concentration mediated by IFN-γ (4 CH) in this study stay within a range that is biologically detectable by the human organism, when at a normal physiological state [28].

The main findings of this study consist in the fact that the tested medicine was able to induce the proliferation and the activation of cells involved in both the innate and the adaptive immune responses, under basal naïve cell culture conditions or under pre-primed conditions mimicked by the T cell receptor (TCR)-activator CD3 signal.

Regarding the immune responses induced under basal conditions, among the treated PBMCs, the subpopulation of human granulocytes has seen the most important increase in their proliferation and activation status (Figure 3B,C, Figures S1 and S2). In addition, we have also observed an enhancement of their functions and, more specifically, an improvement in their phagocytosis capacities (Figure 4B).

The signal transduction pathways induced from the binding of IFN-γ with its receptor and the further activation of GAS has been well studied in granulocytes [29], as well as in other immune cells [1,5,30]. CD69, a cell surface glycoprotein, is one of the target genes whose expression is increased by IFN-γ in granulocytes [29,31]. In line with these findings, homeopathically prepared IFN-γ (4 CH) increased its expression too. Knowing that IFN-γ stimulation and STAT1 activation occur very rapidly, with a peak after only 15–60 min [30,32], the effects observed in IFN-γ (4 CH)-55 min treated-granulocytes (Figure 4A), which have seen an increase in their phagocytic capacities, are not surprising and coherent as a whole. In addition, longer IFN-γ (4 CH)-treatment (48 h) has enhanced the expression of HLA-DR in monocytes/macrophages (Figure 3D), an important molecule induced by IFN-γ, as widely discussed in the introduction section, involved in the specific-antigen presentation machinery, driving the adaptative immune response. HLA-DR has also been slightly upregulated by the tested item in human primary CD14^+^ cells, after a 6-day treatment (Figure 6G, plain-colored histograms). These CD14^+^ cells were cultured for 6 days under basal/standard conditions, classically used to keep macrophages at M0, in the presence of Veh. or IFN-γ (4 CH). In reference to this experiment, performed in CD14^+^ cells, the objective of the study was to investigate how IFN-γ (4 CH) could modulate the M0 macrophages profile towards an M1 status. The M1 profile, which is also referred to as IFN-γ-induced activated-macrophages status, or classical activation/priming, is widely studied, both in vitro and in vivo. M1 macrophages are more aggressive against pathogens and more prone to produce pro-inflammatory mediators, while M2 macrophages play important functions in wound healing, tissue repair and inflammation-resolution. The overall experimental results, presented in Figure 5 and Figure 6, tend to demonstrate that the treatment exerts modulatory effects on M0 macrophages that can adamantly not be compared with the classical and well-recognized effects induced by IFN-γ. The following parts of this discussion aim to analyze the role of each of the assessed cytokines and markers, in regards to their IFN-γ-related immune implication (Figure 5 and Figure 6).

The levels of IFN-γ found in IFN-γ (4 CH)-treated CD14^+^ cells were comparable with the levels found in the IFN-γ (4 CH)-diluted medium alone (Figure 2 and Figure 5C). According to these results, the medicine was not able to influence the secretion of IFN-γ in CD14^+^ cells, under the tested experimental conditions. It can be concluded that the effects and the observed cellular responses are, most likely, mediated by the presence of about 1 to 2 pg/mL of IFN-γ.

In this study, a particular emphasis was made regarding the analysis of the IFN-γ (4 CH)-induced TNF-α modulation, as several pieces of research supported the link between these two factors. For instance, IFN-γ was reported to properly induce TNF-α production in macrophages [33,34]. The same kind of effect, specifically the promotion of TNF-α secretion, has been observed in both IFN-γ (4 CH)-treated and LPS-activated cells. The effect was particularly evident in CD14^+^ cells compared to THP-1 cells (Figure 5B,L,M), probably because the first are primary cells, which are overall more sensitive to environmental changes and different stimuli than cell lines.

In parallel, IFN-γ (4 CH) also promoted the macrophages’ IL-6 secretion (Figure 5F), which is in agreement with the literature, as “super-activated” macrophages cultivated with LPS and IFN-γ together increased the transcriptional activation of pro-inflammatory genes, including TNF-α and IL-6 [30]. Moreover, we also found that IFN-γ (4 CH) increased the secretion of arginase (Figure 5G), an enzyme involved in macrophage inflammatory reactions through the regulation of inducible nitric oxide synthase (iNOS) [35]. Our results are thus in line with those of Ming et al., who reported the implication of arginine-degrading enzyme arginase-2 (Arg II) in the LPS-induced production of pro-inflammatory cytokines by monocytes/macrophages [36], further reinforcing the IFN-γ (4 CH) biological effect on the orientation of the macrophages towards a M1-like phenotype.

Interleukin-12 and IL-23 are two well-known pro-inflammatory cytokines that have largely been described in the literature as important mediators of innate and adaptive immunity [37]. Both share a common subunit, IL12p40 (also known as the heavy chain of the IL-12 heterodimer), that plays a crucial role in the IL-12/IL-23 activation pathway, and whom the gene promoter was reported to be primed by IFN-γ in monocytic cells [38]. More precisely, the authors of this study showed that, in human PBMCs, IFN-γ treatment does not directly induce p40 gene expression and protein production, but it primes monocytic cells to produce them in response to LPS stimulation, which, once again, corresponds to the results we found in the context of this study (Figure 5D,E).

If, from one side, the stimulation of IFN-γ-polarized macrophages with toll-like receptors (TLR) ligands, such as LPS, results in a massive super-induction of inflammatory cytokines and canonical nuclear factor-κB (NF-κB) target genes’ expression, IFN-γ also induces gene-specific refractoriness to anti-inflammatory factors such as IL-10 and IL-4. Macrophages, being “super-activated” by IFN-γ and LPS, increased their responsiveness to pro-inflammatory stimuli and, on the other hand, increased their resistance to anti-inflammatory stimuli [30]. It is also important to highlight that, in the present study, the human CD14^+^-derived macrophages can definitely not be considered as IFNγ-polarized macrophages, nor as M1 macrophages, or even “super-activated” macrophages. Indeed, while activated by LPS, they were treated with 4 CH of IFN-γ, corresponding to about 1 or 2 pg/mL (Figure 2), which does not correspond to the typical doses used to trigger in vitro M1 polarization. IL-10 levels were barely upregulated (by about 4 %), by the tested item under the experimental condition tested (Figure 5H). For what it concerns, the levels in the results of IL-4 were found to be very low, and close to the detection limit in both Veh.- and IFN-γ (4 CH)-treated cells; however, it seems that the tested item could reduce its secretion even more (Figure 5J).

In spite of its pro-inflammatory characteristics, IFN-γ is also able to inhibit the generation of the active form of IL-1β, either through (i) repressing its transcription, as it was demonstrated in murine RAW 264.7 macrophages [39], or (ii) through its ability to repress Nlrp3 inflammasome activation, thus impairing the pro-IL-1β cleavage, as reported in a model of murine bone marrow derived-macrophages [40]. The results obtained in our human CD14^+^-derived macrophages are in accordance with these studies, as we were able to demonstrate that IFN-γ (4 CH) reduced IL-1β secretion by 15% compared with the vehicle-treated cells (Figure 5K). The same trend was obtained regarding the secretion of TARC (Figure 5I), a cytokine implicated in the recruitment of Th2 lymphocytes and the perpetuation of the Th2 responses, abundantly produced by monocytes when cultivated with GM-CSF or IL-3 in addition to IL-4 [41], and produced by murine macrophages when cultivated with IL-4 [42]. Interestingly, the authors of the latter study also reported that IFN-γ was able to inhibit murine TARC mRNA induction by IL-4, when added either before or concomitantly with IL-4 treatment. These data are finally also linked to the inhibitory effect of IFN-γ (4 CH) towards the IL-4 secretion that we presented in Figure 5J, as the effect of IFN-γ towards TARC repression could also, at least partially, be due to its capacity to inhibit IL-4 as well.

In conclusion, regarding the overall data of cytokine expression, the tendency seems to reveal an effect of IFN-γ (4 CH) which can be quite similar (while not in terms of magnitude), to the well-recognized and documented effect induced by the biologically active IFN-γ, with the only exceptions of IL-10 and IL-4 compared to the Veh. control-treated cells. It is, however, worth mentioning that, having been performed only once, all these experiments need to be done again, in order to confirm the observed trends from a statistical standpoint.

The following part of this discussion is dedicated to the cell surface markers’ results, evaluated under basal (without LPS) conditions and in LPS-primed cells.

CD14 has been moderately downregulated by the treatment when compared with the Veh. control (Figure 6B, plain-colored histograms). Even if the magnitude of our results is quite low (about 15% of reduction vs. Veh.), the direction of our observed effects is coherent with previous observations made in human monocytes, which have been cultured with IFN-γ for 48 h, and that have been seen to have reduced their CD14 expression [43]. CD14 is a co-receptor, which substantially contributes to ligand recognition, thus playing an important role in immune responses. CD14 binds different species of LPS, as well as diverse TLR (1, 2, 3, 4, 6, 7, and 9). Indeed, as the CD14’s first recognized biological role is linked to host defenses, it seems to be particularly involved in controlling and regulating the sensitivity of innate immune cells to an LPS-mediated inflammatory stimulus [44]. Once activated, the entire receptor complex, including CD14, is internalized [45], to further induce different pathways and the production of pro-inflammatory regulators (like prostaglandin E2 [PGE2] and IFN-γ). Several pieces of evidence showed that the expression of CD14 is even more downregulated in IFN-γ pre-treated cells followed by LPS stimulation, than in cells receiving only the IFN-γ pre-treatment [43,46]. This mechanism probably aims at reducing the sensitivity to LPS, once the inflammatory reaction is launched. In our study, and contrary to what has been observed by the above-mentioned authors in IFN-γ pre-treated cells, IFN-γ (4 CH) does not seem to influence the CD14 surface levels in LPS-primed cells, compared to the Veh.-treated ones (Figure 6B, squared-pattered histograms). These results, while unexpected, reveal important information as the treatment seems to not reduce the sensitivity of LPS-activated cells. This is interesting to remember in the context of bacterial infection responses and resistance, in which IFN-γ is particularly involved [47].

CD16 has been downregulated by the tested treatment when cells were cultured w/o LPS, while it has been upregulated when cells received both (LPS and 4 CH of IFN-γ) (Figure 6C). The CD16 receptor, also known as FcγRIIIA, is expressed by immune cells such as NK cells and monocytes/macrophages and plays a role in the antibody-dependent cellular cytotoxicity (ADCC) against opsonized (antibody-coated) cells, such as virus-infected cells or cancer cells, through its binding capabilities to the Fc part of the G-Immunoglobulins [48]. The results we obtained here, in the non-LPS-stimulated conditions, were unexpected, as Hartnell et al. reported that IFN-γ induced a dose-dependent increase of the CD16 expression on human eosinophils at concentrations of 100 U/mL and above [49]. Moreover, in LPS-stimulated conditions, Waller et al. observed an ex vivo loss of the CD14^++^/CD16^+^-monocytes population [50], suggesting that LPS could downregulate the expression of this marker. Taken together, our results suggest that, in our model, IFN-γ (4 CH) may need an immune pre-priming, such as LPS for instance, as an additional immune stimulator, to allow an increase in the CD16 expression, as the anti-CD3 was needed to delineate IFN-γ (4 CH)’s effects on the CD69 expression (Figure 7).

CD163 is a specific monocyte/macrophage marker that can be used to discriminate them from the other immune cell populations. While several studies indicated that CD163 may be induced by anti-inflammatory markers such as IL-10, recent evidence revealed that it is wrong to consider it as an M2-specific marker [51]. The first biological role attributed to CD163 is linked to its hemoglobin (Hb) scavenger abilities, as it can bind and remove from the circulation free Hb, known to generate reactive oxygen species (ROS) [52]. This is just one of the mechanisms by which CD163 exerts its anti-inflammatory functions. CD163 is involved in the resolution of inflammation and its presence on the cell surface is fine-tuned in a complex regulatory network. IFN-γ inhibits the upregulation of CD163, probably because it impairs the secretion of IL-10 as well. Interestingly, 4 CH of homeopathically prepared IFN-γ, alone or in association with LPS, induced the CD163 cell surface upregulation, suggesting that, in the tested conditions, the inhibitory effects classically mediated by IFN-γ on CD163 expression did not occur (Figure 6D). This could be explained and linked with the fact that the tested treatment did not reduce IL-10 production (Figure 5H).

The CD200-CD200R axis is considered as an important immunological checkpoint with a pivotal role in the maintenance of immune homeostasis and tolerance [53], as well as in the prevention of autoimmune disease [54,55]. IFN-γ and TNF-α have been shown to induce CD200 expression [56]. In addition, an elevated expression of the receptor CD200R is induced by IL-4 or IL-13, and is associated with an increased number of an alternatively activated (M2a) subtype of human macrophages. These M2a, CD200+, cells exert an anti-inflammatory effect and are involved in Th2 immune responses [57]. Against all expectation, our results of IFN-γ (4 CH)-treated CD14^+^ cells showed that, in both conditions (w or w/o LPS), the tested item has increased the expression of CD200R (Figure 6E). Regarding the fact that CD200-CD200R interactions significantly limit severe autoimmune disorders [54,55], it would be interesting to investigate more about the mechanisms and the clinical potential in this matter.

CD64 is constitutively expressed on monocytes and macrophages and it has a central role in ADCC and in the clearance of immune complexes. It has been shown that IFN-γ is able to upregulate CD64 expression in myeloid-derived cell lines [58], and its rapid clustering on cell membrane with the further immune complex internalization triggers the release of other cytokines, such as TNF-α [59]. Coherent with those findings, our results show that IFN-γ (4 CH) is able to upregulate this M1-macrophage marker under basal conditions (w/o LPS) and under LPS-primed conditions (Figure 6F).

The two final markers studied here are HLA-DR and CD86 (also known as B7-2), both of them being implicated in the regulation of T cell activation. Monocytes/macrophages are immune cells, which, from a biological/functional standpoint, can be considered as cells at the crossroad between the innate and the adaptative responses, thanks to their ability to act as antigen-presenting cells (APC), triggering T cell activation. This specific function is allowed by their capacity to enzymatically digest constituents from microbial origin, such as LPS, for instance, into peptides, and express them on their cell surface, within the groove of an HLA class II molecule, typically HLA-DR [60]. Meanwhile, CD86 is also expressed by APC and displays a dual role in T cell activation, depending on its ligand binding [61]. Interestingly, our results about the expression levels of HLA-DR and CD86, which were both increased by IFN-γ (4 CH) w/o LPS-priming, but decreased by IFN-γ (4 CH) in a LPS-primed environment (Figure 6G,H), resonate with the work of Delneste et al. Their study assessed the effects of IFN-γ on the phenotype of monocytes cultivated during five days in the presence of M-CSF, and showed that the expression level of CD14 was reduced by such treatment, while the ones of HLA-DR and CD86 were increased [62]. Such observations corroborate the tendencies of the results that we presented here, in a non LPS-primed environment (Figure 6B,G,H, plain-colored histograms). Moreover, another study supports the work presented here, as it reports that TNF-α secretion, and the expression levels of HLA-II molecules, in particular, HLA-DR, HLA-DQ and HLA-DP, were reduced in LPS-primed PBMCs compared with control PBMCs [63]. Interestingly enough, by developing a model of LPS activation of murine bone marrow-derived macrophages that generated LPS-activated/acute response (M1) and LPS-tolerized macrophages-states, O’Carroll et al. have highlighted the transcriptional plasticity of macrophages following LPS tolerance [64]. They reported that, even if the CD86 surface expression was significantly increased in cells following acute activation with LPS, the expression of this marker was suppressed during LPS tolerance. Our results, regarding the decreased expression of CD86 induced by IFN-γ (4 CH) in a LPS-primed context (Figure 5H, squared-pattered histograms), may suggest that the tested item could potentially synergize with the LPS-tolerization pathways normally launched by longer LPS exposure.

The information that can be drawn by the above section could be summarized as follows: CD14^+^ cells responded to the tested item by modulating the expression of seven cell surface markers involved in the physiological host immune response (Figure 6). The overall cell phenotype induced by IFN-γ (4 CH), despite its similarity to the M0 profile, and the fact that it is not assimilable to the one of M1 (IFN-γ)-polarized macrophages, has been changed in a manner that may suggest an immune cell activation. In conclusion, the results tend to demonstrate that macrophages respond to IFN-γ (4 CH) stimulus by activating a controlled and moderate response, that, if we suppose that cells are still “prone” to actively respond to other stimuli, because surface markers such as CD163, CD200R and CD64, for instance, were are not downregulated by the treatment. More experiments are still needed to statistically confirm the trends reported here, but overall, this body of data suggest that the low dose homeopathically prepared IFN-γ (4 CH), while corresponding to a biologically available amount of 1 to 2 pg/mL, is able to gently modulate CD14^+^-derived macrophages’ phenotype, with and without LPS co-stimulation.

Interestingly enough, the effects of IFN-γ (4 CH) did not seem to be solely restricted to the activation of macrophage population, as we demonstrated in our next experiment of CD3-pre-primed PBMCs (Figure 7). In this model, our results have indeed shown that IFN-γ (4 CH) led to an increase of about 150% in the CD69 expression of NK cells after having primed the PBMCs with an anti-CD3 antibody (Figure 7B). These data could be put into perspective regarding the fact that activated NK cells are well recognized as early producers of IFN-γ, and they might be one of the first immune cells to even recognize developing tumors, for example [65]. We can hypothesize here that such activation might either be: (i) a direct effect of the IFN-γ (4 CH), or (ii) a direct consequence of the NK cells’ interplay with the activated T cells (CD4^+^ and CD8^+^) (Figure 7C–E), that are all CD3-bearing cells. Such a second activation–model hypothesis could also contribute to synergistically fuel the NK cells’ IFN-γ production that the tested IFN-γ (4 CH) treatment could have biologically initiated in a first place, explaining the impressive magnitude of those cells’ activation (Figure 7B). Be this as it may, the observed increase of the CD69 expression on the B cells, even if its magnitude was one of the lowest (of about 30%, Figure 7F), may also be explained by the interplay between those cells and the CD3-bearing T cells, as the B cell population was discriminated by a CD3- phenotype.

Finally, the last part of our study covered the effect of IFN-γ (4 CH) on endothelial cells, which, alongside their role in vascular hemodynamics, permeability, coagulation and extravasation, are also involved in the sequentiality of the immunity-related processes. Those cells are indeed actively implicated in the elaboration of the immune responses at their early stages [66], and in tissue remodeling and repair at their late stages, during the resolution phase and the healing process aimed at returning to the homeostasis [67]. Their dual role as both (i) inflammation effectors and immune cells recruiters and (ii) important players in tissue repair and homeostasis, is mediated by their ability to express numerous immune-related membrane markers and to secrete large arrays of cytokines and chemokines. In this context, we executed an endothelial cell’s profiling to decipher to what extent the tested unitary MI treatment IFN-γ (4 CH) could influence the expression of four immune-related markers (IFNγ-R1, ULBPs, IL1-R1, and TGFβ-R2). To know the role of IFN-γ and the specific effect induced to those surface markers, the experiment included the treatment with 20 ng/mL of IFN-γ, employed as a reference standard treatment. We observed that all the four surface markers were downregulated by such treatment compared to the control medium only; IFNγ-R1 and ULBPs of about 25% each, while IL1-R1 and TGFβ-R2 were more slightly decreased (about 10% and 5%, respectively) (Figure 8, white histograms vs. dark-green histograms). The same graphs in Figure 8 (grey histograms vs. light-green histograms) show that the expression of these four markers was slightly decreased by IFN-γ (4 CH) compared with the vehicle-treated cells. The results revealed an effect of the unitary-tested MI medicine IFN-γ (4 CH) quite similar (while not in terms of magnitude) to the effect induced by IFN-γ at 20 ng/mL. While they still need to be confirmed from a statistical point of view, these results may confirm that IFN-γ (4 CH) can also affect endothelial cells, and they could be extrapolated at a nice bond between the circulation and the local immune-response sites within the organism.

Overall, from our own viewpoint, and as suggested all along in the result-section of this manuscript, the main limitations of this study lie in the fact that all the presented data came from experiments that have only been performed once, and thus would need to be repeated to add more statistical power to the work presented here. In addition, we only assessed here the biological effect of the homeopathically prepared IFN-γ at the dose of 4 CH, but it is undeniable that, having also included this factor at higher or lower amounts (1 CH vs. 27 CH, for instance) would have been of great value in order to better describe and understand the mechanisms of action of this particular cytokine, when homeopathically prepared, in the context of MI. Regarding the overall experiments performed in the context of this study, our evaluations are limited to the assessment of the biological effects induced by the tested item on immune cells and endothelial cells, and no investigations were made to understand the possible mode of action. Further mechanistic studies, having a different approach, might be conducted with this purpose. Just to give some examples: interactions between the drug and the putative target receptor(s) may be evaluated, or even the presumptive activated signaling pathways involved by those drug–receptor interactions. Finally, the oromucosal delivery of the MIM is another important parameter that has not been taken into account in this in vitro study. Regarding the complexity of the residing cells of the buccal environment and their close interactions with the ones of the immune system, the assessed effects of IFN-γ 4 CH should be reevaluated by using integrated systems that would better mimic the delivery of this factor in vivo. Such perspectives should definitely be addressed in the future, in the hope of penetrating all the facets of MI.

## 4. Materials and Methods

### 4.1. Tested Item and Experimental Control

Biologically active human recombinant (hr)-IFN-γ, produced by Labo’Life Spain (Consell, Spain) in Escherichia coli BL21, was used to manufacture IFN-γ (4 CH), a unitary homeopathic medicinal product consisting of sucrose-lactose pillules, also termed globules, impregnated with ethanolic preparations (96% of ethanol/water) of IFN-γ at 4 CH. The scheme in Figure 1 represents the manufacturing process of IFN-γ (4 CH). Briefly, the hr-IFN-γ starting concentration, obtained by diluting 1 µg of protein in 1 mL of endotoxin-free water, was then used to make the first 1:100 dilution in ethanol 96 per cent V/V. That dilution was further agitated 100 times by a kinetic vertical shaking generated by a dynamizator (Dynamat 50CS, Labotics bvba, Belgium) to make the 1 CH. That process, also called SKP, has been repeated until reaching the desired centesimal dilution used to impregnate the pillules (4 CH, in the current study). Labo’Life employs the SKP process to manufacture MI medicines, both unitary and complex medicines, as previously described [21,22,23,68].

IFN-γ (4 CH) was manufactured and provided by Labo’Life Belgium (Gembloux, Belgium), as well as the vehicle sucrose-lactose pillules (also referred as Veh.) used as experimental control in all the experiments. Those vehicle pills were impregnated with the only ethanolic vehicle preparations (ethanol 96 per cent V/V) used to prepare MI medicines; therefore, those pills lack the active substance. The scheme in Figure 1 also represents the manufacturing process of the Veh. pillules. For all the experiments assessing the in vitro biological effect of IFN-γ (4 CH) on cellular models, either the IFN-γ (4 CH) or the Veh. pillules were freshly diluted in culture medium to reach the final concentration of 11 mM of sucrose-lactose (corresponding to the content of one capsule in 100 mL). Previous experiments and in vitro studies on unitary and complex MI medicines have reported that this excipients’ concentration does not impact cell viability and functions of immune and non-immune cells, and it allows us to measure the active ingredients’ effects [69,70].

### 4.2. Evaluation of IFN-γ Concentration in Basal Medium Containing the Tested Diluted Pillules

RPMI 1640 medium was used to freshly dilute either the IFN-γ (4 CH) or the Veh. pillules at the sucrose-lactose concentrations of 11 mM and 22 mM (corresponding to the content of one capsule in 100 mL and 50 mL, respectively). The experiment was performed in quadruplicates. The concentration of IFN-γ was assessed by ELISA (LegendPlex) according to the manufacturing instructions. The detection limit, which is the lowest possible concentration that can be reliably detected, is 1.04 pg/mL with this kit.

### 4.3. Evaluation of Cell Surface Activation Markers Expression and Proliferation of PBMCs Subpopulations by Flow Cytometry

Freshly isolated PBMCs (from three healthy donors) were cultivated in the presence of either IFN-γ (4 CH) or Veh. during 48 h under classical culture conditions: RPMI 1640 medium added with 2% inactivated human serum, 1 mM non-essential amino acids, 1 mM pyruvate and 10 mM HEPES buffer. Concanavalin A (5 µg/mL) was used as a positive control, regarding its stimulator’s effects towards the induction of the CD69 expression, as shown in Supplementary Figure S1. Immune cells’ populations were delineated based on the following panels in the proliferation experiment: NK cells: CD3^−^, CD11b^−^, CD4^−^, CD8^−^, CD19^−^, CD56^+^, CD14^−^, SSC^low^; monocytes/macrophages: CD3^−^, CD11b^+^, CD4^−^, CD8^−^, CD19^−^, CD56^−^, CD14^+^, SSC^low^; granulocytes: CD3^−^, CD11b^+^, CD4^−^, CD8^−^, CD19^−^, CD56^−^, CD14^−^, SSC^high^; B cells: CD3^−^, CD11b^−^, CD4^−^, CD8^−^, CD19^+^, CD56^−^, CD14^−^, SSC^low^; T cells: CD3^+^, CD11b^−^, CD4^+^, CD8^+^, CD19^−^, CD56^−^, CD14^−^, SSC^low^; CD4^+^ T cells: CD3^+^, CD11b^−^, CD4^+^, CD8^−^, CD19^−^, CD56^−^, CD14^−^, SSC^low^; CD8^+^ T cells: CD3^+^, CD11b^−^, CD4^−^, CD8^+^, CD19^−^, CD56^−^, CD14^−^, SSC^low^. In the granulocyte’s activation experiment, the CD69 expression was assessed amongst the granulocyte population characterized by the following panel: CD3^−^, CD11b^+^, CD4^−^, CD8^−^, CD19^−^, CD56^−^, SSC^high^. In the monocytes/macrophages’ activation experiment, the HLA-DR expression was assessed amongst the population characterized by the following panel: CD3^−^, CD11b^+^, CD14^+^, SSC^low^. The cells were labeled and analyzed by flow cytometry on a BD FACS Canto II, configuration 4/2/2.

### 4.4. Evaluation of Cell Surface Activation Markers Expression of PBMCs Subpopulations by Flow Cytometry, under CD3 Pre-Primed Condition

Freshly isolated PBMCs were cultivated in within the same classical culture conditions described in the previous paragraph, in the presence of 0.5 µg/mL bottom-coated OKT3 antibody (anti-CD3). Concanavalin A (5 µg/mL) was used as a positive control, regarding its stimulator’s effects towards the induction of the CD69 expression, as illustrated in the graphs of the Supplementary Figure S2. In the activation assessment experiment, the immune cells’ populations were delineated based on the following panels: NK cells: CD3^−^, CD11b^−^, CD4^−^, CD8^−^, CD19^−^, CD56^+^, SSC^low^; B cells: CD3^−^, CD11b^−^, CD4^−^, CD8^−^, CD19^+^, CD56^−^, SSC^low^; T cells: CD3^+^, CD11b^−^, CD4^+^, CD8^+^, CD19^−^, CD56^−^, SSC^low^; CD4^+^ T cells: CD3^+^, CD11b^−^, CD4^+^, CD8^−^, CD19^−^, CD56^−^, SSCl^ow^; CD8^+^ T cells: CD3^+^, CD11b^−^, CD4^−^, CD8^+^, CD19^−^, CD56^−^, SSC^low^. The CD69 expression level was assessed among each of the above-mentioned subpopulation.

### 4.5. Phagocytosis

Granulocytes were isolated from total peripheral blood, after Ficoll^®^ gradient separation. The cells were grown in RPMI-1640 medium supplemented with L-glutamine 2 mM, Penicillin 100 U/mL-Streptomycin 100 μg/mL and BSA 0.1% at 37 °C and 5% CO_2_. The experiment was performed in 96-well plates; IFN-γ (4 CH) or the positive control fMLP 10 µM (used as a phagocytosis inducer) were preincubated with the cells during 10 min at 37 °C. In order to include the proper experimental control, the vehicle was run in parallel too. Fluorescent beads (Molecular Probes™ FluoSpheres™ Carboxylate-Modified Microspheres, 1.0 μm, yellow-green fluorescent 505/515) were then added and incubated with the cells for 45 min. Untreated non-bead-incubated cells were used as a negative control. All the conditions were performed in triplicate. In Figure 4A is represented the experimental scheme. After the incubation period, cells were rinsed in PBS/BSA 0.1% and centrifugated. Acquisitions were realized with 10,000 cells/replicate on a BD FACSVerseTM cytometer. Regarding that the fluorescence-increase in the FITC channel is proportional to the phagocytosed beads (emission beads wave length = 515 nm), the results were first expressed as FITC-positive percentages, then expressed in a percentage of the vehicle-treated cells, set as 100%.

### 4.6. Macrophage Cell Surface Marker Expression and Cytokine Secretion Evaluation

CD14^+^ cells were freshly isolated from PBMC thanks to the Miltenyi kit (130-050-201). Briefly, the cells were then seeded at the density of 50,000 cells/well in 96-well plates on Day 0 and were cultivated in RPMI 1640 supplemented with 2% inactivated human serum, 1 mM non-essential amino acids, 1 mM pyruvate, 2 mM L-glutamine, 10 mM HEPES buffer and 50 ng/mL M-CSF. Cells were treated with vehicle or IFN-γ (4 CH) from Day 1 until Day 7, both medium and treatments being renewed on Day 3 and Day 5. The experimental scheme is shown in Figure 5A.

For cytokine secretion assessment, the cells were stimulated with LPS on Day 6 (100 ng/mL) and harvested on Day 7. Supernatants were collected after cells centrifugation, and cytokine measurement was directly performed by ELISA (LegendPlex) on fresh supernatants. The assessed secreted-cytokine’s panel included IFN-γ, IL-23, TNF-α, IL12p40, IL-6, arginase, IL-10, TARC, IL-4, and IL-1β. For the macrophage cell surface marker experiment, the cells were stimulated or not with LPS on Day 6 (100 ng/mL), harvested, labeled and analyzed by flow cytometry on Day 7, on a BD FACS Canto II, configuration 4/2/2. The intensity of the staining was measured as a median fluorescence intensity (MFI) value. The cell viability was appraised based on a gating of the NIR-Zombie positive cells (data not shown). The M0 and M1 macrophage populations were discriminated based on their expression of CD14/CD64/CD86 and identified as follows: M0: CD14^+/−^, CD64^+^, CD86^+^; M1: CD14^++^, CD64^+++^, CD86^++^. The expression of the cell surface markers CD14, CD16, CD163, CD200R, CD64, HLA-DR and CD86 was evaluated. Because only one experiment was conducted, no statistical inference has been performed. The results of the cell markers and cytokines secretion are presented as the mean ± SD of *n* = 3 replicates per condition.

### 4.7. Evaluation of Cytokine Secretion in THP-1 Cells

Human monocytic THP-1 cells were obtained from ATCC (Manassas, VA, USA) and cultured in RPMI 1440 supplemented with 10% FCS and antibiotics. 500,000 cells/well were seeded in 24-well plates and the cells were pre-treated with the freshly diluted pillules, Veh. or IFN-γ (4 CH) for 30 min, and stimulated with LPS (1 μg/mL) for either 24 h or 30 h. The supernatants were collected and stored at −80 °C until use. TNF-α was measured by ELISA assays according to the instruction of the manufacturer (R&D Systems, Minneapolis, MN, USA).

### 4.8. HUVECs Immunoprofiling by Flow Cytometry

Primary human umbilical vein endothelial cells (HUVECs) (Passage 4) were seeded at 5000 cells/well in 96-well plate in ECGM medium (Promocell, Heidelberg, Germany) supplemented with 2% fetal bovine serum (FBS), and were grown for 4 days before adding IFN-γ (4 CH) or vehicle to the medium. Commercial human recombinant IFN-γ (20 ng/mL) (# ref 130-096-481, Miltenyi Biotec B.V. and Co. KG, Bergisch Gladbach, Germany) was used as a positive control and run in parallel, in the same experiment. The medium alone was run to include the negative control as well. The cells were treated for 48 h (with a medium/treatment renewal after 24h) before multiparametric immunostaining and flow cytometry analysis. The intensity of the staining was measured as median fluorescence intensity (MFI) value. One experiment was performed in triplicate for each condition. The viability (data not shown) and the expression levels of ULBPs, IL-1R1, TGFβ-R2 and IFNγ-R1 were assessed.

### 4.9. Statistical Analysis

The graphs in the figures were performed with GraphPad Prism, Version 9.3.1 for Windows (GraphPad Software Inc., San Diego California USA, accessed on the 3rd of January 2022). The authors have followed the recent recommendations that encourage performing descriptive statistics instead to make statistical inferences when the number of independent values is small. Indeed, no statistical inference has been performed to analyze the results of the in vitro studies presented here. In order to show the results in a transparent manner, when data were obtained from only one experiment, replicates were plotted together with the mean ± SD in the graphs (Figure 2, Figure 4, Figure 5 and Figure 6 and Figure 8). Regarding PBMC experiments, the data are represented as the mean ± SEM of values obtained from *n* = 3 healthy donors (Figure 3 and Figure 7). The data of each donor are also provided and represented in scatter plot graphs that allow us to visualize the individual experimental replicates together with the mean ± SD (Supplementary Figures S1 and S2).

## 5. Conclusions

In the context of the fundamental research of homeopathically prepared signaling molecules, and particularly in pursuing the research about efficacy and mode of action of MI medicines, our findings, while limited to only one cytokine and only one centesimal Hahnemannian dilution (4 CH), bring more knowledge about their biological effects and their possible mechanism of action. For the first time, after dilution of the IFN-γ (4 CH)-bearing pillules, the concentration of the active substance in the medium has been evaluated and measured. In addition, preclinical in vitro drug response experiments have been performed in a wide panel of immune cells, under basal or pre-primed conditions, showing how IFN-γ (4 CH)-treated cells reply to the treatment by enhancing their proliferative or phagocytic capacities, by modulating the expression of several markers, as well as their cytokines secretion. It can finally be concluded that the tested unitary MI medicine may be an interesting immunostimulatory and immunomodulatory drug and deserves further investigation.

## Figures and Tables

**Table 1:** IFN-γ dosage (expressed in pg/mL), within the IFN-γ (4 CH)-pillules, when diluted in RPMI medium to reach the sucrose-lactose’s excipients concentrations of 11 mM and 22 mM.

	IFN-γ (4 CH) Sucrose-Lactose Pillules Concentrations	
Parameters (pg/mL)	11 mM	22 mM
Mean	1.63	1.91
SD	0.43	0.69
Median	1.6	1.72
Max	2.12	2.74

SD: standard deviations; Max: maximal value obtained within the technical quadruplicate of the tested pillules.

## Data Availability

Not applicable.

## Supplementary Materials

The following supporting information can be downloaded at: https://www.mdpi.com/article/10.3390/ijms23042314/s1.

## Abbreviations

AA: amino acids; BSA: bovine serum albumin; ADCC: antibody-dependent cellular cytotoxicity; APC: antigen-presenting cells; CH: centesimal Hahnemannian; FBS: fetal bovine serum; FCS: fetal calf serum; GM-CSF: granulocyte-macrophage colony-stimulating factor; Hb: hemoglobin; HLA: human leukocyte antigen; HUVECs: human umbilical vein endothelial cells; IFN-γ: interferon-γ; IFNγ-R1: interferon-γ receptor 1; IL-1β: interleukin-1β; IL1-R1: interleukin-1 receptor 1; JAK1/2: janus kinase 1/2; LD: low dose; LPS: lipopolysaccharide; M1: M1 macrophage; MCH: major histocompatibility complex; M-CSF: macrophage colony-stimulating factor; MI: micro-immunotherapy; NK: natural killer; NKT: natural killer T cells; PBMCs: peripheral blood mononuclear cells; PBS: phosphate buffer saline; P/S: penicillin/streptomycin; ROS: reactive oxygen species; SKP: serial kinetic process; STAT1: signal transducer and activator of transcription 1, TCR: T cell receptor; TGF-β: transforming growth factor-β; TGFβ-R2: transforming growth factor-β receptor 2; TLR: Toll like receptors; TNF-α: tumor necrosis factor-α; ULBP: UL16-binding proteins.
