# Supplementary material for: The Unitary Micro-Immunotherapy Medicine Interferon-γ (4 CH) Displays Similar Immunostimulatory and Immunomodulatory Effects than Those of Biologically Active Human Interferon-γ on Various Cell Types
Source: Int J Mol Sci. 2022 Feb 19;23(4):2314. doi: 10.3390/ijms23042314 (PMC8879050; doi:10.3390/ijms23042314)
Supplement: Supplementary file 1 [file ijms-23-02314-s001.zip › ijms-1595607-supplementary.pdf]

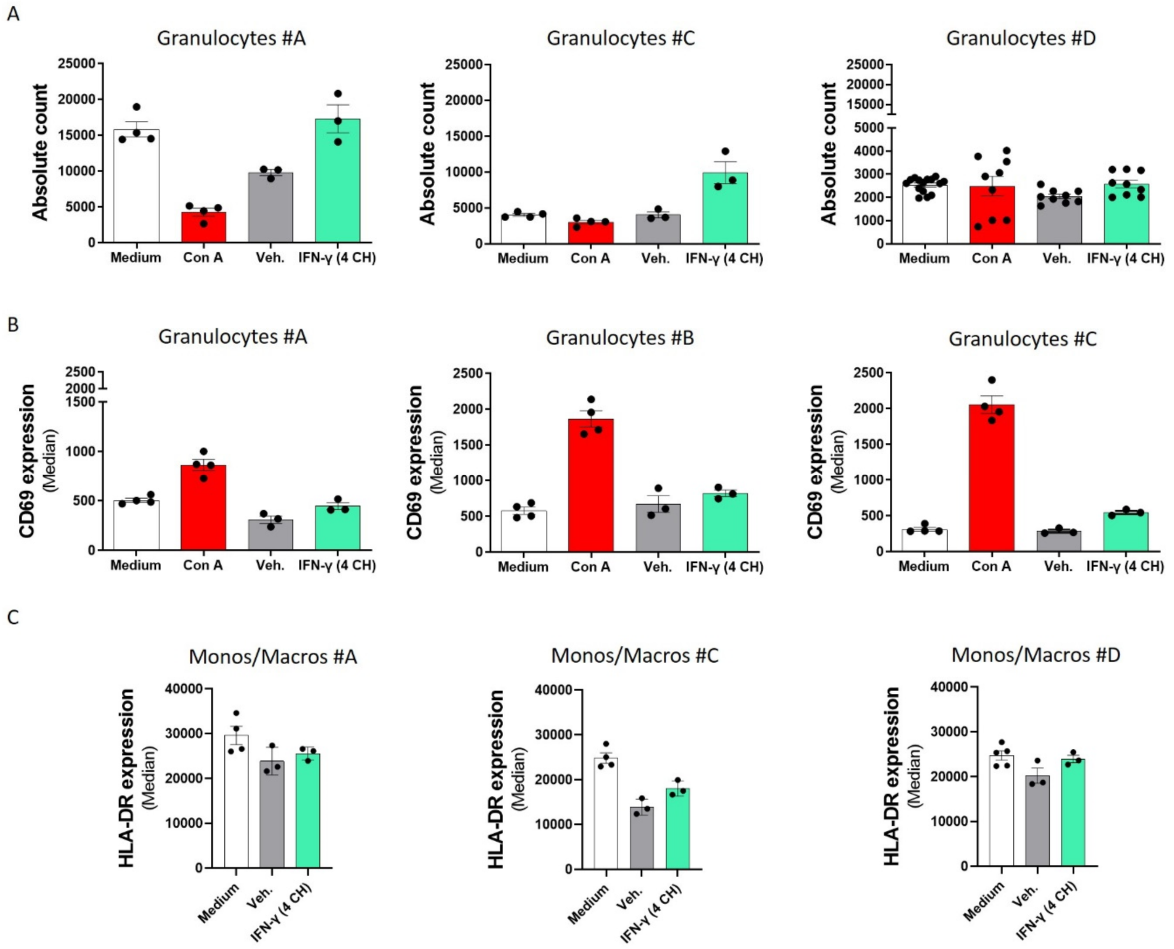

Supplementary Figure S1. IFN-γ (4 CH) increases the proliferation of human granulocytes in vitro, as well as the activation of granulocytes and monocytes/macrophages. Human PBMCs from healthy donors (referred as donors #A, #B, #C and #D) were cultivated during 48 hours in classical culture conditions (Ct medium) in presence of IFN-γ (4 CH) or Vehicle (Veh.). Concanavalin A (Con A) at 5 µg/mL was used as a positive control, regarding its stimulator's effects towards the induction of the CD69 expression. (**A**) The total number of granulocytes was assessed by flow cytometry; this cell subpopulation being discriminated from the other PBMCs according to the expression of the markers referred in Section 4. The experiment has been done once and each histogram represents the mean ± SEM of the cell count of technical triplicates obtained for each individual donor. (**B**) The expression of the activation marker CD69 was assessed by flow cytometry within the granulocytes subpopulation. The experiment has been done once and each histogram represents the mean ± SEM of the CD69 expression in technical triplicates obtained for each individual donor. (**C**) The expression of the activation marker HLA-DR was assessed by flow cytometry within the monocytes/macrophages, those cells being discriminated from the other PBMCs according to the expression of the markers referred in Section

4. The experiment has been done once and each histogram represents the mean ± SEM of the HLA-DR expression in technical triplicates obtained for each individual donor.

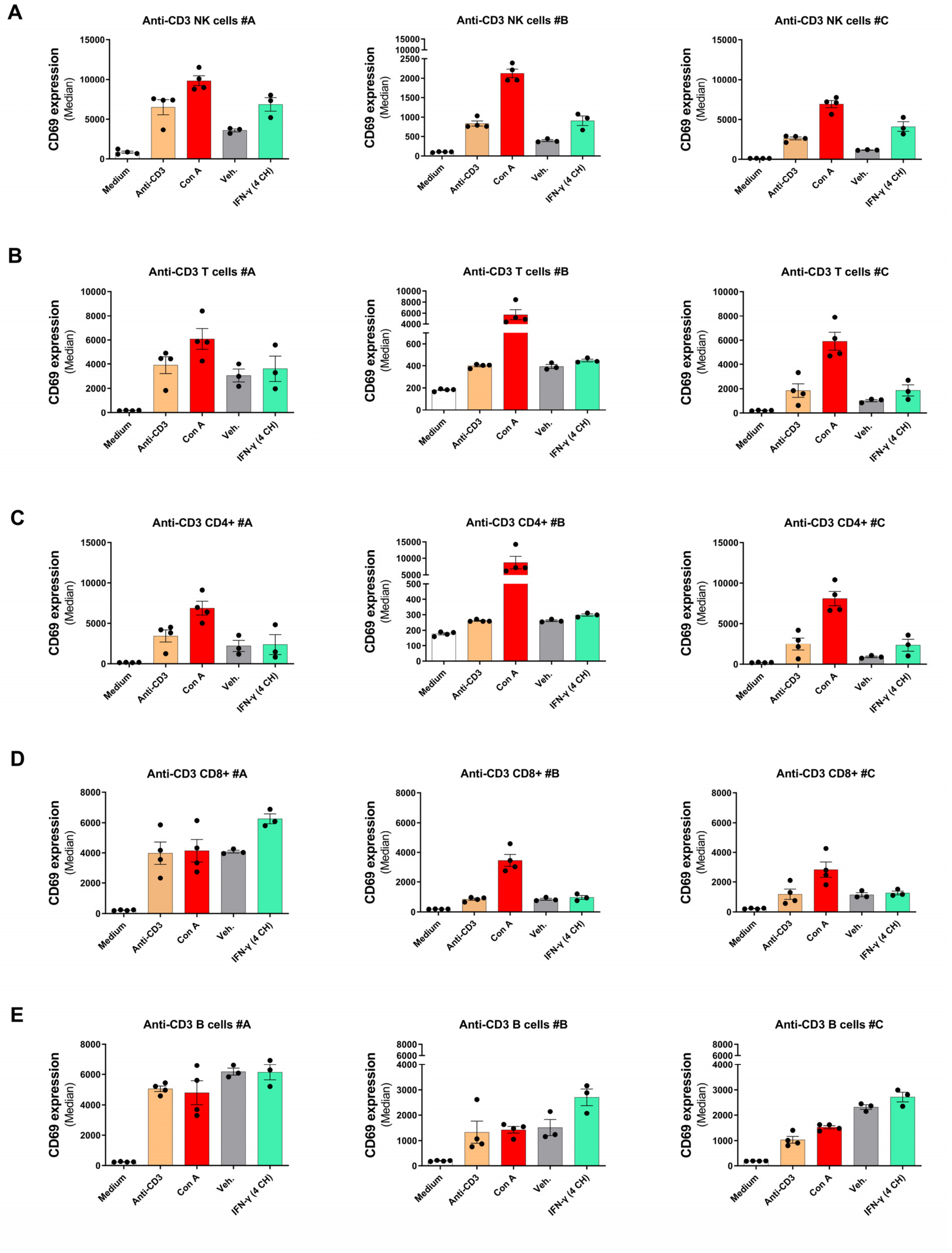

Supplementary Figure S2. IFN-γ (4 CH) enhances the activation of CD3-pre-primed immune cells in vitro. Human PBMCs from three healthy donors (referred as donors

#A [left panel], #B [middle panel], and #C [right panel]) were cultivated during 48 hours in classical culture conditions plus coated-anti-CD3 antibody at 0.5 μg/mL (thereafter referring as "anti-CD3"), in presence of IFN-γ (4 CH) or Vehicle (Veh.). Concanavalin A (Con A) at 5 μg/mL was used as a positive control, regarding its stimulator's effects towards the induction of the CD69 expression. The CD69 expression was assessed by flow cytometry within: (**A**) NK cells, (**B**) T cells, (**C**) $CD4^+$ T cells, (**D**) $CD8^+$ T cells, and (**E**) B cells. Each cell subpopulation was discriminated from the other PBMCs according to the expression of the markers referred in Section 4. The experiment has been done once and each histogram represents the mean ± SEM of the CD69 expression in technical triplicates obtained for each individual donor.
